# Supplementary material for: Analysis of PPARγ Signaling Activity in Psoriasis
Source: Int J Mol Sci. 2021 Aug 10;22(16):8603. doi: 10.3390/ijms22168603 (PMC8395241; doi:10.3390/ijms22168603)
Supplement: Supplementary file 1 [file ijms-22-08603-s001.zip › Supplemental materials_Analysis of PPARg signaling activity in psoriasis/Pathway models/Models images and html files/PPARG expressed in cells assotiated with psoriasis/226606.html]

Th2 cell


# CellType Th2 cell

|  |  |
| --- | --- |
| URN | urn:agi-ncimcelltype:C0242633 |
| Total Entities | 47 |
| Connectivity | 3584 |
| Name | Th2 cell |

---

|  |  |
| --- | --- |
| ChildConcepts | T helper cell function |
|  | T-helper 2 cell differentiation |
|  | T-helper lymphocyte infiltration |
|  | Th2 cell phenotype |
|  | Th2 cell development |
|  | Th2 cell migration |
|  | Th2 cell repertoire |
|  | Th2 cell polarity |
|  | Th2 cell priming |
|  | Th2 cell population |
|  | Th2 cell fate commitment |
|  | Th2 cell formation |
|  | Th2 cell survival |
|  | Th2 cell proliferation |
|  | Th2 cell ratio |
|  | Th2 cell homing |
|  | Th2 cell growth |
|  | Th2 cell chemotaxis |
|  | Th2 cell apoptosis |
|  | Th2 cell fate |
|  | Th2 cell fate determination |
|  | Th2 cell proliferative response |
|  | Th2 cell death |
|  | Th2 cell clonal anergy |
|  | Th2 cell adhesion |
|  | Th2 cell rolling |
|  | Th2 cell homeostasis |
|  | Th2 cell damage |
|  | Th2 cell autophagy |
|  | Th2 cell extravasation |
|  | Th2 cell destruction |
|  | Th2 cell motility |
|  | Th2 cell fate specification |
|  | Th2 cell developmental process |
|  | Th2 cell sequestration |
|  | Th2 cell contact |
|  | Th2 cell viability |
|  | Th2 cell transepithelial migration |
|  | Th2 cell phagocytosis |
|  | Th2 cell transendothelial migration |
|  | Th2 cell interaction |
|  | Th2 cell division |
|  | Th2 cell distribution |
|  | Th2 cell transdifferentiation |
|  | Th2 cell cytotoxicity |
|  | Th2 cell recognition |
|  | Th2 cell disruption |

---

|  |  |
| --- | --- |
| Pathway | STAT3 Facilitates the Function of Treg Cells and Cancer Progression |
|  | IL13/STAT6/ZEB1 Signalling Promotes Cancer Metastatic Progression |
|  | Tumor Infiltrating Macrophages in Cancer Progression and Immune Escape |
|  | PPARG expressed in cells assotiated with psoriasis |
|  | Overview. Diseases of the skin (chapter 11) |
|  | 1\_Th2 cell response in asthma |
|  | 2\_3\_Eosinophilia and neutrophilia in asthma: Neutrophil chemotaxis and activation |

---

|  |  |
| --- | --- |
| MedScan ID | 10003435 |

---

|  |  |
| --- | --- |
| Alias | helper cell type 2 |
|  | T helper 2 Th2 cells |
|  | T-helper cell (Th) type 2 cells |
|  | T helper 2 Th2 cell |
|  | helper T cell type 2 |
|  | T helper 2 cell |
|  | T helper Th2 cells |
|  | Th2 cell |
|  | type 2 helper T cells |
|  | Th2 cells |
|  | T-helper cell (Th) type 2 cell |
|  | type 2 helper T cell |
|  | T helper type 2 |
|  | T helper Th2 cell |
|  | helper T cells type 2 |
|  | T helper 2 cells |
|  | T helpers type 2 |
|  | helper cells type 2 |

---

|  |  |
| --- | --- |
| NCIm ID | C0242633 |

---
